# The Role of the NMDA Receptor in the Anticonvulsant Effect of Ellagic Acid in Pentylenetetrazole-Induced Seizures in Male Mice

**DOI:** 10.1155/2022/9015842

**Published:** 2022-05-11

**Authors:** Mohammad Rahimi-Madiseh, Zahra Lorigooini, Shakiba Nasiri Boroujeni, Marziyeh Taji, Hossein Amini-Khoei

**Affiliations:** Medical Plants Research Center, Basic Health Sciences Institute, Shahrekord University of Medical Sciences, Shahrekord, Iran

## Abstract

**Methods:**

In this experimental study, 64 mice were divided into 8 groups and received the following: normal saline; EA at doses of 6.25, 12.5, and 25 mg/kg; NMDA agonist at a dose of 75 mg/kg; NMDA antagonist (ketamine) at a dose of 0.5 mg/kg; an effective dose of EA plus NMDA agonist; and a subeffective dose of EA plus ketamine. We induced seizure using intravenous administration of PTZ. 60 minutes before induction of seizure, drugs were administrated. Duration lasts to seizure-induced was measured. Finally, the gene expression of NMDA receptor subunits (*Nr2a* and *Nr2b*) was assessed in the prefrontal cortex.

**Results:**

Results showed that EA increased the seizure threshold and decreased the expression of Nr2a and Nr2b. We determined that ketamine potentiated and NMDA attenuated the effects of subeffective and effective doses of EA.

**Conclusion:**

EA probably via attenuation of the NMDA-R pathway possesses an anticonvulsant effect in PTZ-induced seizure in mice.

## 1. Introduction

Seizure is a common disease in the world [1]. The seizure occurs when an abnormal electrical activity manifests in the central nervous system (CNS). Various etiologies are involved in the pathophysiology of seizure [2]. According to the researcher's findings, 30 to 40% of patients are resistant to all treatments and do not respond adequately to the standard anticonvulsants [3]. Despite the introduction of new drugs in recent decades, these are accompanied by several side effects [4]. Studies have shown that changes in gabaergic and glutamatergic synaptic transmission have a pivotal role in seizure development. Seizures occur when the activity of excitatory neurotransmitters (glutamate) increases or the activity of inhibitory neurotransmitters (GABA) decreases [5].

Glutamate is a nonessential dicarboxylic amino acid in various brain structures [6]. As a significant stimulatory neurotransmitter, glutamate participates in several physiological functions such as brain development, synaptic flexibility, memory, and learning [5, 7]. An increase in the glutamate concentration in the synaptic space and extracellular fluid leads to neuron damage [8]. N-Methyl-D-aspartate (NMDA) is one of the ionotropic glutamate receptors. Activation of NMDA receptor causes accumulation of calcium ions in neurons [9, 10]. Studies have shown an increase in the activation and expression of NMDA receptor subunits during epilepsy [11]. In this regard, it has been determined that agents that antagonized the activity of NMDA receptors exerted significant anticonvulsant effects [12, 13]. Previous studies have demonstrated that activation of NMDA receptors increases the severity of the seizure and diminishes the anticonvulsant effect of some effective agents [14]. Studies in animal models have demonstrated that seizures differentially change the expression of NMDA receptor subunits, including NR2a and NR2b, in the brain [15]. It has been determined that blockade of either NR2a or NR2b subunits of NMDA receptors decreases status epilepticus-induced neuronal cell death [16]. In this regard, researchers have demonstrated that seizure increased the expression of NR2a and NR2b in the brain [17].

Recently, the central concept in seizure management research is finding agents with neuroprotective activities [9, 10]. Ellagic acid (EA), with a molecular weight of 302 g/mol, is a polyphenolic compound with antioxidant properties found in fruits like pomegranate, raspberry, and berry [18]. Previous studies have reported several pharmacological properties for EA, including antibacterial, anti-inflammatory, immunoregulatory, and antitumor effects [19–22]. It has been suggested that EA exhibits a neuroprotective effect and protects the brain from oxidative and inflammatory challenges [11, 23, 24]. Preclinical examinations determined antidepressant-like effects for EA [25, 26].

Considering the role of glutamate and NMDA receptors in the pathophysiology of seizure and several pharmacological effects of EA, especially its neuroprotective effects, in this study, we aimed to evaluate the possible involvement of NMDA-R in the anticonvulsant effect of EA in pentylenetetrazole- (PTZ-) induced seizures in male mice.

## 2. Material and Methods

### 2.1. Study Design

64 male NMRI mice were kept under standard laboratory conditions (12-hour light/dark cycle, 22 ± 2°C, and free access to water and food). All stages of the present experimentation were carried out following the regulations of the university and the Guide for the Care and Use of Laboratory Animals of the National Institutes of Health (Ethics code: IR.SKUMS.REC.1397.312) and Guide for the Care and Use of Laboratory Animals (8th edition, National Academies Press). Full efforts were made to diminish animals' use and improve their wellbeing. Mice were randomly divided into 8 groups (*n* = 8). Group 1 received normal saline (considered as a control group), groups 2-4 received EA at doses of 6.25, 12.5, and 25 mg/kg, respectively, group 5 received NMDA agonist at the dose of 7.5 mg/kg, group 6 received NMDA antagonist (ketamine) at the dose of 0.5 mg/kg, group 7 received the effective dose of EA plus NMDA agonist, and group 8 received the subeffective dose of EA plus ketamine. All drugs were administrated intraperitoneally (i.p.). 60 minutes after the treatments, pentylenetetrazole (PTZ) was injected intravenously at an 80 mg/kg dose to induce seizures. The dose and time of drug administrations were chosen based on previous studies and our pilot study [27–29].

### 2.2. Induction of Seizures by PTZ

In order to inject the PTZ, a needle gauge 30 was attached to the mice's tail vein. After fixing the mice's tail, the PTZ (0.5%) was injected at a 1 mL/min rate by a seizure pump. The injection stopped as soon as the clonus of the anterior limb was seen. The minimum dose of PTZ for seizure was considered the dose of seizure threshold. In this method, the seizure threshold was dependent on the PTZ dose and was related to time. PTZ was injected intravenously at a dose of 80 mg/kg 60 minutes after treatments [27].

### 2.3. Real-Time PCR Analysis for Expression of NMDA Receptors in the Prefrontal Cortex

At the end of the study, animals were sacrificed, the prefrontal cortex was isolated, and the gene expression of NMDA receptor subunits (*Nr2a* and *Nr2b*) was examined by real-time PCR. Firstly, total RNA using TRIzol reagent (Invitrogen) was extracted from the prefrontal cortex. Alterations in the mRNA levels of genes were determined using qRT-PCR after the reverse transcription of 1 *μ*g of RNA from each sample using the PrimeScript RT reagent kit (Takara Bio, Inc., Otsu, Japan). qRT-PCR was done on a light cycler device (Roche Diagnostics, Mannheim, Germany) using SYBR Premix Ex Taq technology (Takara Bio). Thermal cycling conditions included an initial activation step for 30 s at 95°C afterward 45 cycles, a denaturation step for 5 s at 95°C, and a combined annealing/extension step for 20 s at 60°C. Melting curve analysis was performed to certify whether all primers yielded a single PCR product. The genes and their primers are listed in Table 1. *H2afz* was used as a house-keeping gene (normalizer), and alterations in the expression of each target mRNA in comparison with B2m were measured based on the 2-*ΔΔ*Ct relative expression formula, as described in our previous publication [30, 31].

### 2.4. Statistical Analysis

Statistical analysis of data was performed using GraphPad Prism 8 software. One-way ANOVA was used to determine the significant differences between the treatments, and the Tukey post hoc test compared the means. Data were recorded as mean ± standard deviation, and *P* < 0.05 was considered statistically significant.

## 3. Results

### 3.1. Effects on the Seizure Threshold

**Figure 1:**
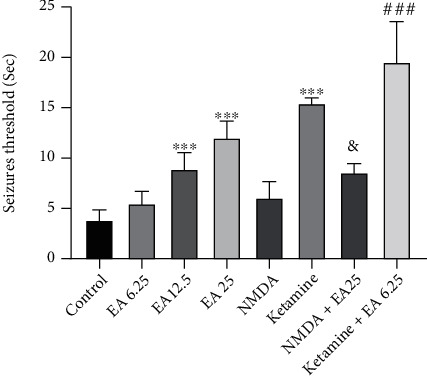
Seizure threshold (second) in the experimental groups. Data are reported as mean ± standard deviation from 8 animals and statistically analyzed by one-way ANOVA and Tukey post hoc test. ^∗∗∗^*P* < 0.001 in comparison to the control group (saline-treated), ^###^*P* < 0.001 in comparison to the group that received EA at a dose of 6.25 mg/kg, and ^&^*P* < 0.05 in comparison to the group that received EA at a dose of 25 mg/kg. EA: ellagic acid.

The results showed (Figure 1) that EA at doses of 12.5 and 25 mg/kg significantly increased the seizure threshold in comparison to the control group (*P* < 0.001). We showed that ketamine significantly increased the seizure threshold compared to the control group (*P* < 0.001). Administration of a subeffective dose of EA (6.25 mg/kg) plus ketamine significantly increased the seizure threshold in comparison to the group that received a subeffective dose of EA alone (*P* < 0.001). Furthermore, coinjection of NMDA agonist plus an effective dose of EA (25 mg/kg) significantly decreased the seizure threshold in comparison to the group that received an effective dose of EA alone (*P* < 0.05).

### 3.2. Effect on Gene Expression of NMDA Receptors in the Prefrontal Cortex

**Figure 2:**
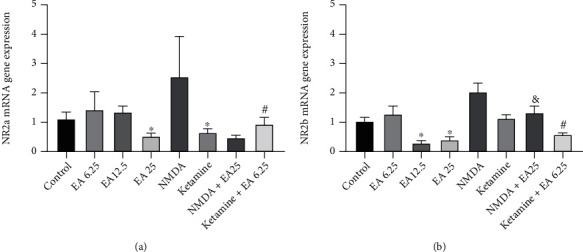
Expression of NMDA receptor genes in the prefrontal cortex in the experimental groups: (a) *Nr2a*; (b) *Nr2b*. Data are reported as mean ± standard deviation and statistically analyzed by one-way ANOVA and Tukey post hoc test. ^∗^*P* < 0.05 in comparison with the control group (saline-treated), ^#^*P* < 0.05 in comparison with the group that received EA at the dose of 6.25 mg/kg, and ^&^*P* < 0.05 in comparison with the group that received EA at the dose of 25 mg/kg. EA: ellagic acid.

According to the results (Figure 2(a)), EA at a dose of 25 mg/kg significantly decreased the gene expression of *Nr2a* in comparison to the control group (*P* < 0.05). Ketamine as an NMDA receptor antagonist significantly reduced the gene expression of *Nr2a* in comparison to the control group (*P* < 0.05). We found that coadministration of ketamine plus the subeffective dose of EA (6.25 mg/kg) significantly decreased the gene expression of Nr2a in comparison to the group that received a subeffective dose of EA alone (*P* < 0.05).

The present study results showed that EA at doses of 12.5 and 25 mg/kg significantly decreased the gene expression of Nr2b compared to the control group (*P* < 0.05, Figure 2(b)). Simultaneous injection of the subeffective dose of EA (6.25 mg/kg) with ketamine significantly reduced the expression of the *Nr2b* gene compared to the group that received the subeffective dose of EA alone (*P* < 0.05). Moreover, coinjection of the effective dose of EA (25 mg/kg) plus NMDA agonist significantly increased the *Nr2b* gene in comparison to the group that received an effective dose of EA alone (*P* < 0.05).

## 4. Discussion

The present study showed that EA possessed the anticonvulsant effect and increased the threshold of PTZ-induced seizures in mice. We demonstrated that inhibition of NMDA receptor using ketamine potentiated the anticonvulsant effect of a subeffective dose of EA. Findings determined that coadministration of NMDA agonist with the effective dose of EA attenuated the anticonvulsant effect of an effective dose of EA. Our data showed that EA decreased the gene expression of *Nr2a* and *Nr2b* subunits of NMDA receptors in the prefrontal cortex.

It has been well-determined that the glutamatergic system and NMDA receptors are involved in the pathophysiology of seizures [32]. In this concept, it has been determined that in subsequent seizures, the concentration of glutamate increased in the synaptic space and extracellular fluid, which through its excitatory toxicity leads to neural damages [33]. The molecular basis of this cytotoxicity is not well understood; however, there is some agreement that the accumulation of calcium ions within neurons following activation of NMDA receptors leads to neuronal damages [34, 35]. Activation of NMDA receptors induces long-term alteration of synaptic connections and alteration of neuronal circuits, which may involve the pathogenesis of seizure [16]. It has been determined that NMDA receptor antagonists exerted anticonvulsant effects [12, 13]. Moreover, previous studies have demonstrated that agonists of NMDA receptors increase the severity of seizures and diminish the anticonvulsant effect of some anticonvulsants [36].

In this regard, past studies have shown that changes in the expression of NMDA receptor subunits, including NR2a and NR2b, play a vital role in the pathophysiology of seizures [37]. In this concept, animal studies showed that seizures increased the expression of *Nr2a* and *Nr2b* subunits of the NMDA receptors in the brain [38]. Previous studies have demonstrated that selective NR2b blockers significantly attenuate seizures and increase seizure threshold [39]. In a study by Mathern et al., on patients with seizures, an increase in mRNA levels and change in the composition of ionotropic glutamate receptors in the temporal lobe of the brain were observed. It has been suggested that these changes may play a role in neuronal stimulation, neuronal synchronization, and seizures [40]. In our study, in line with aforementioned previous studies, induction of seizures by PTZ leads to an increase in gene expression of NMDA receptor subunits including *Nr2a* and *Nr2b* in the prefrontal cortex.

EA is a polyphenolic compound found in some fruits [41]. Ample evidence reported neuroprotective effects for EA in various neurological models [42–44]. Lorigooini et al. showed that EA by attenuating the NMDA receptors possessed antidepressant-like effects in mice [45]. In a study conducted by Girish et al., it has been determined that EA via activation of the gabaergic system improves learning and memory [46]. Previously, it has been demonstrated that EA possessed anti-inflammatory effects and reduced oxidative stress [47]. Dhingra and Jangra showed that EA could attenuate the seizures in PTZ-induced seizures in mice [48].

Furthermore, ameliorative effects of EA on maximal electroshock and PTZ-induced seizures in mice have been reported [49]. Recently, EA has been shown that modulating oxidative stress and inflammatory cytokines ameliorates PTZ-induced seizures in mice [50]. However, the exact mechanisms involved in the anticonvulsant effect of EA have not been fully determined. In line with the aforementioned studies, we found that EA increased the seizure threshold in PTZ-induced seizures in mice.

This study found that following treatment with EA, the gene expression of *Nr2a* and *Nr2b* subunits of NMDA receptors in the prefrontal cortex significantly decreased. They were indicating that attenuation of NMDA receptors may mediate the anticonvulsant effects of EA. To examine this hypothesis, we treated mice with an EA plus agonist and/or antagonist of the NMDA receptor. Findings showed that ketamine (the NMDA antagonist) potentiated while NMDA agonist attenuated the anticonvulsant effects of subeffective and effective doses of EA, respectively. These results determined that the NMDA receptor, partially at least, is involved in the anticonvulsant effects of EA.

## 5. Conclusion

Our results showed that EA exerts an anticonvulsant effect. We found that the anticonvulsant effect of EA, partially at least, mediated through attenuation of NMDA receptors.

## Figures and Tables

**Table 1:** Sequences of primers.

Sequence	Name
CTCAGCATTGTCACCTTGGA	*Nr2a*-F
GCAGCACTTCTTCACATTCAT	*Nr2a*-R
CTACTGCTGGCTGCTGGTGA	*Nr2b*-F
GACTGGAGAATGGAGACGGCTA	*Nr2b*-R
TCATCGACACCTGAAATCTAGGA	*H2afz*-F
AGGGGTGATACGCTTTACCTTTA	*H2afz*-R

## Data Availability

The data used to support the findings of this study are available from the corresponding author upon request.
